# Engineering Porous Poly(lactic acid) Scaffolds with High Mechanical Performance via a Solid State Extrusion/Porogen Leaching Approach

**DOI:** 10.3390/polym8060213

**Published:** 2016-05-31

**Authors:** Hua-Mo Yin, Jing Qian, Jin Zhang, Zai-Fu Lin, Jian-Shu Li, Jia-Zhuang Xu, Zhong-Ming Li

**Affiliations:** College of Polymer Science and Engineering, State Key Laboratory of Polymer Materials Engineering, Sichuan University, Chengdu 610065, China; scugfzyhm@163.com (H.-M.Y.); JingQ_scu@163.com (J.Q.); zhangjin500782@126.com (J.Z.); Linzfscu@163.com (Z.-F.L.); jianshu_li@scu.edu.cn (J.-S.L.)

**Keywords:** solid state extrusion, poly(lactic acid), scaffolds, mechanical reinforcement, porosity

## Abstract

A knotty issue concerning the poor mechanical properties exists in the porogen leaching approach to porous scaffolds, despite its advantage in tuning pore structure. To address this hurdle, solid state extrusion (SSE) combined with porogen leaching was utilized to engineer porous scaffolds of poly(lactic acid) (PLA). Advances introduced by poly(ethylene glycol) (PEG) caused the PLA ductile to be processed and, on the other hand, enabled the formation of interconnected pores. Thus, a well-interconnected porous architecture with high connectivity exceeding 97% and elevated porosity over 60% was obtained in the as-prepared PLA scaffolds with the composition of NaCl higher than 75.00 wt % and PEG beyond 1.25 wt %. More strikingly, the pore walls of macropores encompassed countless micropores and rough surface topography, in favor of transporting nutrients and metabolites as well as cell attachment. The prominent compressive modulus of the PLA scaffolds was in the range of 85.7–207.4 MPa, matching the normal modulus of human trabecular bone (50–250 MPa). By means of alkaline modification to improve hydrophilicity, biocompatible porous PLA scaffolds exhibited good cell attachment. These results suggest that the SSE/porogen leaching approach provides an eligible clue for fabricating porous scaffolds with high mechanical performance for use as artificial extracellular matrices.

## 1. Introduction

A foremost strategy to substitute autografts is the utilization of three-dimensional porous scaffold, which serves as a temporary template for cell adhesion, proliferation, along with differentiation to form a functional tissue [1,2] and effectively overcomes the problems of lacking availability and allografts suffering from existent complications. Of particular importance is the fabrication method, since it endows scaffolds with the required physical and biological properties for tissue regeneration [3,4,5,6]. A wide spectrum of techniques have been proposed to engineer porous scaffolds, including fiber bonding [7], porogen leaching [8,9,10,11,12,13,14], emulsion freeze drying [15], gas foaming [16,17], thermally induced phase separation [18], electrospinning [19,20], and rapid prototyping [21]*.* Among them, porogen leaching is perceived as the most expedient and economic access to porous structures. The tunable pore shape, size, and porosity are easily acquired through control over particle parameters such as appearance, dimension, and fraction. However, despite its distinct convenience, potential toxicity from residual organic solvents poses an intrinsic limitation to bone repair [22].

To pursue a hazarding solvent-free route, a combination of melt processing with innoxious porogen leaching seems to be straightforward, convenient, and efficient. Compression molding in conjunction with water-soluble salt removal has become a versatile way to devise tissue engineering scaffolds. The obtained scaffolds are constitutive of uniformly interconnected and independently controllable pore networks [10]. This strategy applies equally well to extrusion and injection molding [23,24]. Unfortunately, in spite of the feasibility, porous scaffolds prepared by these approaches always suffer from poor mechanical properties, which can hardly fulfill the requirements of the host bone tissue engineering [25]. Here, it is emphasized that the scarcity in mechanical properties of artificial scaffolds is also a common and intractable issue faced by other fabrication techniques. Even worse, potential thermal degradation derived from a high processing temperature further deteriorates the mechanical properties. Therefore, ameliorating the mechanical performance of bone regeneration scaffolds remains challenging.

Strong interest in constructing high strength and modulus polymer products results in the emergence of solid state extrusion (SSE), which garners superior self-reinforced materials without thermal degradation [26,27,28,29,30]. The principle of SSE is to compulsively extrude polymeric billets through convergent dies just below the melting point. During SSE, shear deformation induces the formation of well-defined fibrillar structures in the extruded materials, consequently leading to a remarkable increase in mechanical properties. One representative study registered that flexural strength and modulus increased 1.7-fold and 3.0-fold for SSE poly(lactic acid) (PLA) compared to the melt-extruded counterpart, respectively [31].

The facile yet versatile SSE approach exhibits technical advances in comparison to other methods, such as avoiding organic solvents, repelling molecular weight degradation, and improving mechanical properties. In this contribution, we attempted to fabricate PLA porous scaffolds by combining SSE and hydrosoluble sodium chloride (NaCl) leaching. PLA, a biodegradable, biocompatible, and absorbable polyester, is recognized as one of the most attractive scaffold materials [32] for being successfully implanted *in vivo*, such as in human heart-blood vessels [33,34]. Nevertheless, innate poor toughness of PLA is destructive to processability, which is an obstacle to the wider use in the fields of tissue engineering [35]. With an accent on optimizing workability of PLA, biocompatible poly(ethylene glycol) (PEG) was introduced as an effective plasticizer for PLA [36,37,38,39,40]. Meanwhile, water-soluble PEG could serve as polymeric porogen and was expected to cooperate with NaCl to promote the generation of porous structures. Our results demonstrated that the SSE/porogen leaching technique is a feasible strategy to fabricate porous PLA scaffolds with a high compressive modulus, an interconnected interior structure, as well as a good cell adhesion capacity for tissue engineering.

## 2. Materials and Methods

### 2.1. Materials

PLA with l-lactide content of 99.8% was supplied by Zhejiang Hisun Biomaterials Co., Ltd. (Taizhou, China). The *M*_n_ and *M*_w_ were 9.0 × 10^4^ and 19.5 × 10^4^ g·mol^−1^, respectively. PEG (trade name Carbowax) with *M*_w_ = 3350 g·mol^−1^ was purchased from Dow Chemical Company (Midland, MI, USA). NaCl as particulate porogen was purchased from Chengdu Kelong Chemical Reagent Factory (Chengdu, China). The particle size was in the range of 25–74 μm. All chemicals for cell experiment were provided by the Chengdu Baoke Biotechnology Co., Ltd. (Chengdu, China).

### 2.2. Preparation of PLA/PEG/NaCl Mixtures

Ternary blends of PLA/PEG/NaCl were melt-compounded using a torque rheometer (XSS-300, Shanghai Kechuang Rubber Plastic Mechanical Equipment Co., Ltd., Shanghai, China). PLA, PEG, and NaCl with the formulae as listed in Table 1 were dried at 45 °C for 48 h in vacuum prior to blending. The mixing process was conducted at 190 °C with a rotation speed of 30 rpm for 7 min. Subsequently, the blends were shattered into small granules via a universal high-speed smashing machine (Beijing Kewei Yongxing Instrument Co., Ltd., Beijing, China). Binary blends of PLA/NaCl could not be prepared due to inferior processability. Herein, the code P_75-20_ denotes the sample composed by 75.00 wt % NaCl, 5.00 wt % PEG, and 20.00 wt % PLA, where the relative mass faction (MR) of PEG in the polymeric components is 20 wt %. For brevity, the rest is deduced by analogy.

### 2.3. Preparation of Porous PLA Scaffolds

**Figure 1:**
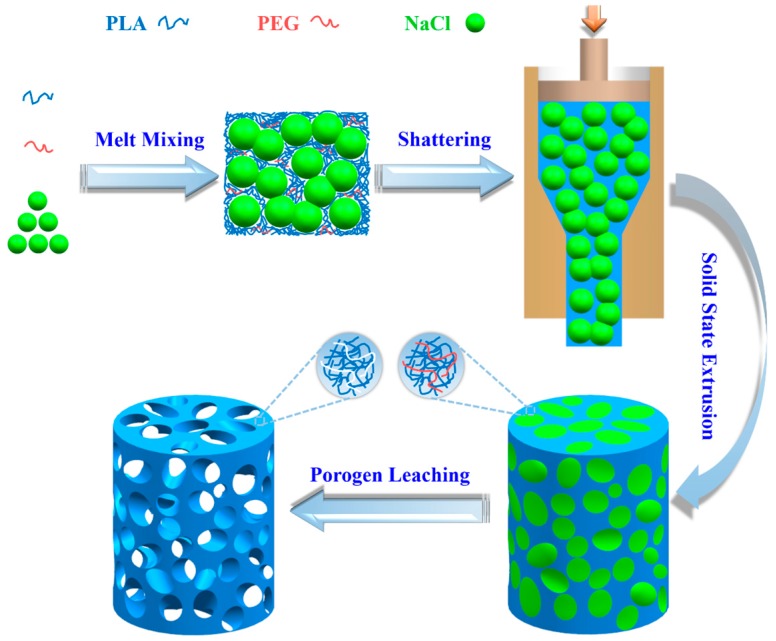
Schematic of fabricating porous PLA scaffolds. The size of NaCl ranges from 25 to 74 μm, and the diameter of as-prepared porous scaffolds is 10 mm.

A home-made SSE apparatus was employed to prepare porous PLA scaffolds. Diameters of cylindrical channel in the barrel and the conical die were 20 and 10 mm, respectively, corresponding to the extrusion draw ratio of 4. A cylindrical billet of the PLA/PEG/NaCl mixture was first annealed at 185 °C for 15 min by compression molding and slowly cooled to 55 °C at a rate of 3 °C·min^−1^. Then, the billet was heated to 155 °C and kept for 10 min to reach a thermal balance, and was then extruded through the die. Meanwhile, the drawing rate was maintained slowly enough to guarantee ample time for the deformation of the billet and to gain an impeccable cylindrical extrudate. Following the machining of entitative specimens, cylinders and discs were immersed in distilled water at 37 °C for a continued 36 h to leach out the NaCl and PEG. Finally, the prepared PLA scaffolds were dried at 37 °C in a vacuum to a constant weight. Figure 1 illustrates the preparation process of porous PLA scaffolds as previously described.

### 2.4. Residual Mass, Connectivity, and Porosity Measurement

Residual weight percentage of NaCl in the PLA scaffolds was determined by thermogravimetric analysis (Q600, TA Instruments Inc., New Castle, DE, USA). The sample was heated at a constant rate of 20 °C·min^−1^ from 30 to 600 °C. In order to confirm that the residual weight at 600 °C solely resulted from NaCl, the weight loss curve of pure PEG was present as a control. The connectivity of porous PLA scaffolds was calculated by the following equation:

(1) $$Connectivity=(1-\frac{\omega_{\mathrm{residual}}}{\omega_{\mathrm{original}}})\times 100\%,$$

where ω_residual_ and ω_original_ are the residual and original weight fraction of NaCl, respectively.

Porosity of PLA scaffolds was measured by a gravimetric method according to the following equation:

(2) $$Porosity=(1-\frac{\rho }{\rho_{c}})\times 100\%,$$

where apparent density (ρ) of PLA scaffolds is defined as the mass divided by the volume of porous PLA scaffolds, while ρ_c_ represents the density of compact PLA (1.27 g·cm^−3^). Here, the theoretical values of porosity by assuming the complete extraction of PEG and NaCl are tabulated in Table 1.

### 2.5. Scanning Electron Microscopy (SEM)

Morphology observation was examined by a field-emission SEM (Inspect F50, FEI, Hillsboro, OR, USA) at an acceleration voltage of 20 kV. The observed specimens were fractured after freezing in liquid nitrogen for ~30 min and then sputtered a thin golden layer. Pore size was quantified by measuring 200 randomly distributed pores for 3 times with image analysis software of Nano Measurer 1.2.0 (provided by Laboratory of Surface Chemistry and Catalysis, Department of Chemistry, Fudan University, Shanghai, China).

### 2.6. Differential Scanning Calorimetry (DSC)

Melting behavior was determined by a DSC instrument (Q2000, TA Instruments, New Castle, DE, USA). The samples (5–6 mg) were heated from 40 to 190 °C at a constant rate of 10 °C·min^−1^ under a nitrogen flow rate of 50 mL·min^−1^. The crystallinity (*X*_c_) was calculated by *X*_c_ = [(∆*H*_m_ − ∆*H*_cc_)/∆*H*_0_] × 100%, where Δ*H*_m_ and Δ*H*_cc_ delegate melting enthalpy and cold crystalline enthalpy, respectively. Δ*H*_0_ is the melting enthalpy of 100% crystalline PLA, 93.7 J·g^−1^ [41].

### 2.7. Mechanical Properties

Compressive property was carried out at ambient temperature using a universal mechanical testing machine (Model 5567, Instron Instruments, Norwood, MA, USA) at a crosshead speed of 1 mm·min^−1^ and with a 1-kN load cell. Cylindrical samples with a diameter of 10 mm and a height of 9–10 mm were used for the measurement. An average value of 5 specimens in each group was presented with standard deviation.

### 2.8. Water Contact Angle Analysis

Wettability was qualitatively examined via the measuring water contact angle (WCA) using a contact angle goniometer (DA 30, Krüss, Hamburg, Germany) equipped with internal image analysis software. Distilled water (2 μL) was dropped on the surface of dry PLA scaffolds at room temperature and the wetting process was recorded using a high-speed digital camera. Average WCA was obtained by performing on five random regions in a specimen.

### 2.9. Cytotoxicity Test

*In vitro* cell cytotoxicity experiment was performed using an extract solution method to assess material cytotoxicity. Rat bone marrow stromal cells (BMSCs) were seeded on a 96-well plate (3000 cells·well^−1^) and incubated in Dulbecco’s modified eagle medium (DMEM) containing 10% fetal bovine serum and penicillin-streptomycin (100 μg·mL^−1^) at 37 °C in a humidified 5% CO_2_ atmosphere for 24 h. Then, the medium was completely removed and extract solution (100 μL) was added to each well. After 24 h of incubation, the cell viability was analyzed by the 3-(4,5-dimethylthiazol-2-yl)-2,5-diphenyltetrazolium bromide (MTT) colorimetric assay. Specifically, 10 μL of MTT solution (5 mg·mL^−1^ in the final concentration) was added to each well and incubated for 5 h. Afterwards, supernate was removed, and 100 μL of dimethyl sulfoxide (DMSO) was put into each well to dissolve formazan crystals. The absorbance at 492 nm was measured by a microplate reader (KHB ST-360, Shanghai Kehua Bio-engineering Co., Ltd., Shanghai, China). The experiment of each specimen was performed six times.

### 2.10. Cell Adhesion

Cell attachment was used to examine the cytocompatibility of porous PLA scaffolds. Sterilized discs were seeded with BMSCs (10,000 cells·well^−1^) in 48-well plates and cultured in the above DMEM condition for 24 h. For observing cell morphology, the samples were removed from the 48-well plates and rinsed three times with a phosphate-buffered saline (PBS). Then, 50 mL of the fluorescein diacetate (FDA; Sigma, St. Louis, MI, USA) was immediately added to each sample and incubated in darkness at 37 °C for 15 min. Subsequently, all samples were rinsed with PBS and observed by an inverted fluorescence microscopy (Eclipse TS100, Nikon, Tokyo, Japan). To quantitatively evaluate cell adhesion *via* CCK-8, discs after 24 h of incubation were transferred to other 48-well plates. The DMEM media (0.2 mL) and CCK-8 solution (20 μL) were added to each well, and transferred into a 96-well plate after 1 h of incubation. The microplate reader was utilized to measure the absorbance at 450 nm.

### 2.11. Statistical Analysis

All data were expressed as mean ± standard deviation. Statistical significance was examined by single-factor analysis of variance (ANOVA). A value of *p* < 0.05 was considered statistically significant.

## 3. Results and Discussion

### 3.1. Structural Integrity

**Figure 2:**
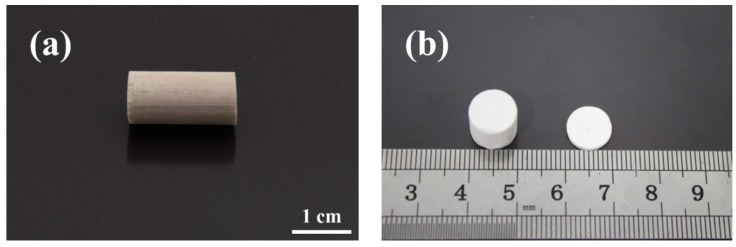
Digital photos of porous PLA scaffolds for P_80-20_: (**a**) before porogen leaching and (**b**) after machining and porogen leaching.

An integrated structure is a necessary prerequisite for PLA scaffolds to prevent premature collapse and to promote bone tissue regeneration *in vivo*. As evidenced by the existing literatures, inclusion of NaCl component depresses the chain mobility of PLA and results in inferior flowability [40]. Our preliminary exploration also found that there were visible cracks on the surface of PLA/NaCl extrudates; subsequently, the binary blends collapsed irreparably during the leaching process due to structural defect. In the melting extrusion of polymer/particulate blends, a tradeoff between flowability and porosity should be pursued, both of which are associated with particulate content. When particulate content was below 70.00 wt %, the extrudates usually possessed low porosity and poor pore interconnectivity [42]. Further increases in particulate content cause a loss of flowability. As a concession, high temperature curing was used to assure the structure integrity and high porosity but at the expense of molecular weight and mechanical properties [23]. Pleasingly, in our study, even the as-prepared scaffold materials containing NaCl amounts more than 80.00 wt % were extruded favorably, as presented in Figure 2a. This is because the presence of PEG in the ternary blends is conducive to a decrease in viscosity and the improvement in flowability [40]. Further evidence for good structural integrity can be furnished from leached P_80-20_, as visualized in Figure 2b. Porous cylinder and disc have the same diameter with the produced extrudate after machining bulk SSE specimens followed by the removal of NaCl particles and PEG in distilled water. The SSE/porogen leaching approach is flexible enough to engineer porous scaffolds with various shapes to meet different application requirements.

### 3.2. Residual Mass and Connectivity

**Figure 3:**
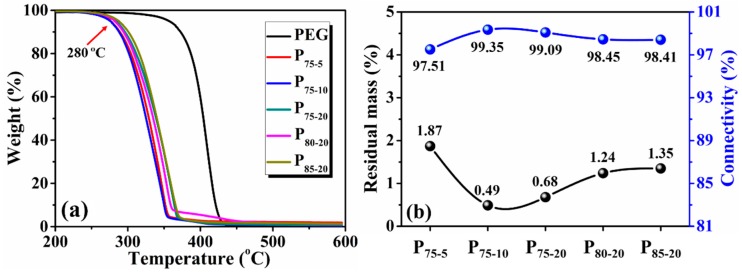
(**a**) TGA curves of porous PLA scaffolds and PEG control; (**b**) Residual mass and connectivity as a function of sample composition.

TGA was performed to determine the residual mass of NaCl and the connectivity of porous scaffolds. To exclude the inference of PEG in residual weight, the TGA curve of pure PEG was presented as a control (Figure 3a). In the temperature range of 350–450 °C, PEG undergoes primary thermal decomposition and shows 100% weight loss. This indicates that the ultimate residual mass of porous PLA scaffolds is solely from undissolved NaCl moiety. The remaining mass percent of all samples is less than 2%, verifying a complete particulate leaching of NaCl (Figure 3b). Regarding the sample comprised of 75.00 wt % NaCl (P_75-5_, P_75-10_ and P_75-20_), the residual mass decreases with PEG concentration. In addition, the onset decomposition temperature at 5% weight loss of all the as-prepared PLA scaffolds remains nearly constant at about 280 °C (Figure 3a). No significant difference in the initial decomposition temperature suggests scarce change of molecular weight in SSE PLA scaffolds.

Consistent with a previous report [40], rinsing out water-soluble PEG is in favor of improving pore interconnectivity (Figure 3b). All the porous PLA scaffolds exhibit high connectivity in excess of 97%. This is indicative of the easy formation of an interconnected porous architecture by dint of the SSE/porogen leaching approach [43]. Such an observation is notably superior to prior studies, where NaCl particles, even at the loading of 80.00 wt %, usually became trapped within the matrix, resulting in unsatisfactory connectivity because of a lack of access to water [24]. The interconnected channels induced by the leaching of PEG is conducive to NaCl removal, leading to the escalation of connectivity. Therefore, the recommended method provides a pacific yet simple cue for preparing an interconnected structure.

### 3.3. Morphology and Structure

**Figure 4:**
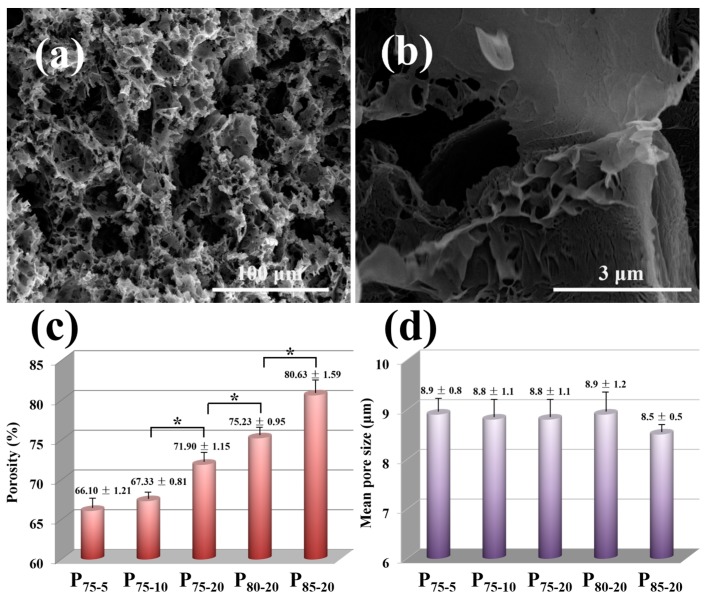
SEM images of (**a**) fracture surface and (**b**) pore wall of P_80-20_. (**c**) Porosity of PLA scaffolds for all sample compositions. (**d**) Mean pore size of scaffolds acquired from SEM images. * indicates *p* < 0.05.

SEM images of P_80-20_ are shown as an example to depict the inner structure of the porous PLA scaffolds. A highly interconnected porous architecture in three dimensions appears after the removal of porogen (Figure 4a), in line with the TGA results. The similar structure is observed in other samples except P_85-20_, in which the collapse of pore structures is observed, likely due to the discontinuous pore connections induced by high NaCl content and inferior processability (Figure S1). Note that lots of randomly distributed micropores throughout pore walls might be derived from the removal of PEG and diminutive NaCl particles (Figure 4a). It should be addressed that small channels could not only afford high interconnectivity among pores, as demonstrated in Figure 3b, but also enable the transport of nutrients and metabolites in scaffolds during cell culturing [44]. Furthermore, leaching hydrosoluble PEG engenders a rough pore wall surface (Figure 4b), which has a positive effect on promoting cell attachment and proliferation [45]. Similar to Ghosh’s report [46], permeable pores located in pore walls accelerate NaCl removal by the dissolution of PEG. On the other hand, an essential factor of porosity [47] shows a monotonical increase accompanied by the increase in PEG and NaCl (Figure 4c), reaching from 66.10% for P_75-5_ to 80.63% for P_85-20_. As expected, the porosity of fabricated PLA scaffolds coincides with the theoretical value (Table 1), further supporting the idea that the full removal of porogen leads to the formation of interconnected pore structure (Figure 3b). Reminiscent of the basic porosity requirement (60%) of tissue engineering, the as-prepared porous scaffolds tender enough space to boost cell proliferation and growth [48]. Average pore size estimated from SEM images shows an approximately identical value of 9 μm, which has little relation to PEG and NaCl content (Figure 4d). A smaller pore size than the particle size of primitive NaCl is attributed to the fragmentation of bulky NaCl caused by intensive shear field and smash action in the processing. Relatively uniform pore distribution and micron-scale pore size ensure enough space for cell infiltration [48].

### 3.4. Thermal Behavior

**Figure 5:**
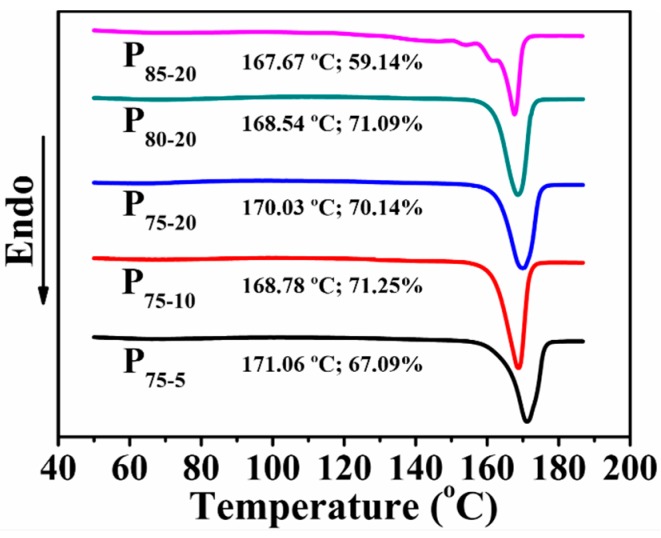
DSC melting thermograms of scaffolds for different compositions.

Since *X*_c_ of scaffolds strongly influences cell attachment [49], thermal properties of porous PLA scaffolds were further evaluated. As given in Figure 5, *X*_c_ and melting point (*T*_m_) are detected from the first heating curves of the PLA scaffolds with varied compositions. Note that all porous PLA scaffolds with the exception of P_85-20_ display relatively high *X*_c_. Upon the introduction of PEG, the increased mobility of PLA chains contributes to the formation of the regular structure [40]. Such well-arranged crystalline structure is envisioned to enhance resistance on compressive deformation. An account of decreased *X*_c_ in P_85-20_ is that high NaCl content impedes PLA chain mobility and thereby prohibits crystallization. Besides, only a single *T*_m_ from P_75-5_ to P_80-20_ reflects a homogenous structure of PLA crystals. In contrast, multiple *T*_m_s emerge in the 1st heating curve of P_85-20_. Crystal transformation from α-form to β-form disclosed in our previous work may be the reasonable interpretation [50].

### 3.5. Compressive Properties

**Figure 6:**
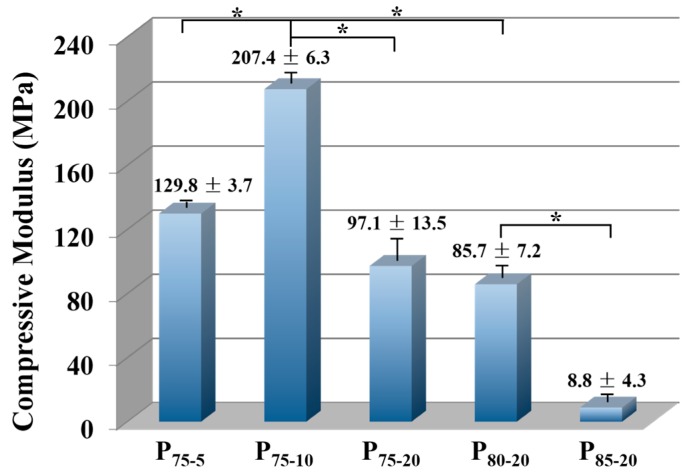
Compressive modulus of scaffolds for different compositions. * indicates *p* < 0.05.

The matched mechanical properties of porous scaffolds are crucial for loading in early transplants and promoting bone reparation [44]. Based on the current perspective, ideal scaffolds require suitable mechanical properties for sustentation so as to match the native tissue. However, it is rarely realized in the porous scaffolds. As seen in Figure 6, the compressive modulus first increases with PEG and then decreases from P_75-10_ to P_85-20_, reaching up to 207.4 MPa. Such a high value in the compressive modulus is closely related to the structural transformation induced by the SSE. By driving PLA/PEG/NaCl blends to convergent dies, a more compact and robust polymeric framework is formed. The compressive modulus of porous scaffolds also relies on the structural parameters, such as porosity, pore size, and autologous structure. Since P_75-5_ and P_75-10_ have similar porosity and mean pore size (Figure 4c,d), the obvious improvement in compressive modulus could probably be ascribed to the stable internal structure [36]. The increase in PEG content from 1.25 to 2.50 wt % makes the PLA chain easier to migrate, generating a neater structure, as corroborated by *X*_c_ (Figure 5). As a result, P_75-10_ is capable of withstanding outside force more effectively. Upgrading porosity to porous PLA scaffolds with identical mean pore size and structural regularity can lead to a more open pore structure (Figure 4c,d and Figure 5) and hence impair the compressive modulus from 207.4 MPa in P_75-10_ to 85.7 MPa in P_80-20_. In comparison with the PLA scaffolds with analogous porosity once reported (8.4–35.0 MPa) [51,52], the value of compressive modulus in the present work exhibits an over twofold increase, even reaching an unprecedented level [53]. More importantly, the compressive moduli of all sample compositions except P_85-20_ situates themselves in a middle and even upper level of the normal modulus (50–250 MPa) of human trabecular bone [54]. These results suggest that porous scaffolds gained via the SSE/porogen leaching method are suitable for bone repair. Taking the comprehensive properties of porous PLA scaffolds into account, P_80-20_ is regarded as the most appropriate candidate to further evaluate its biocompatibility.

### 3.6. Surface Wettability

**Figure 7:**
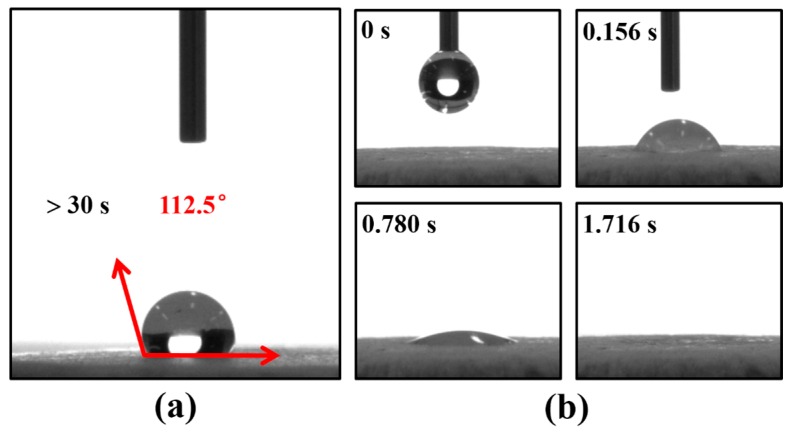
Water contact angle (WCA) of scaffolds for P_80-20_ (**a**) before and (**b**) after alkaline modification.

Since surface wettability of scaffolds intensively affects cell attachment, hydrophilicity of the PLA scaffolds was accessed by measuring the water contact angle (WCA). An average WCA of 112.5° for P_80-20_ is observed in Figure 7a, which is due to the hydrophobicity of PLA. As described by Lin *et al.*, a hydrophobic surface precludes cell adhesion in cell culture [55]. Expecting a promotion effect on cell attachment and growth, alkaline modification was introduced to improve wettability [14]. As shown in Figure 7b, the rapid water infiltration process (1.716 s) indicates that hydrophilicity of P_80-20_ is improved by hydrophilic modification overtly.

### 3.7. Cytotoxicity Test and Cell Adhesion

**Figure 8:**
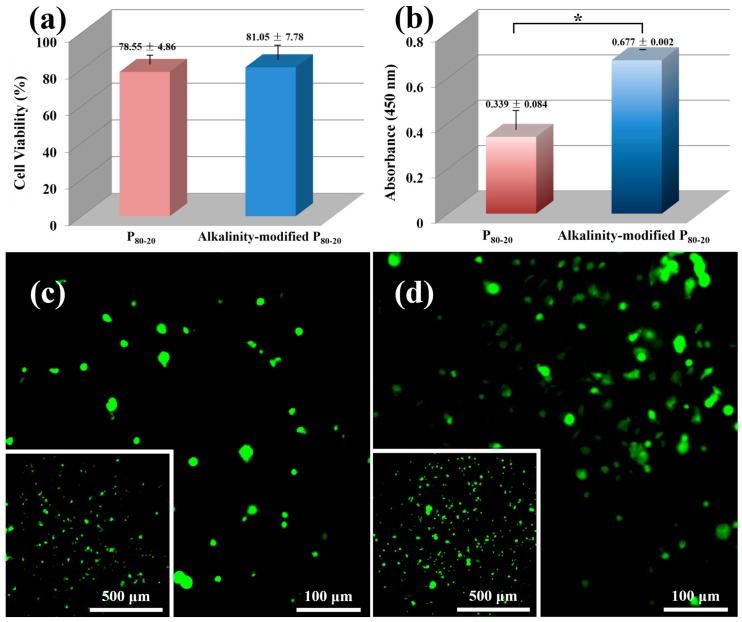
(**a**) Cytotoxicity of the scaffolds by extract solution analysis. (**b**) Cell adhesion of the scaffolds cultured for 1 day. Fluorescence image of bone marrow stromal cells (BMSCs) after adhesion on (**c**) P_80-20_ and (**d**) alkalinity-modified P_80-20_ (live cells are stained in green). The insets of (**c**) and (**d**) present low-magnification image of cell morphology. * indicates *p* < 0.05.

Rudimentary cytotoxicity test by the extract solution method was adopted to evaluate the cytotoxicity of scaffolds. According to ISO10993-5 (2009), both P_80-20_ and alkalinity-modified P_80-20_ show the cell viability to be more than 70% (Figure 8a), manifesting that the porous scaffolds are nontoxic and biocompatible. Qualitative analysis of the BMSC attachment on PLA scaffolds is reflected in fluorescence micrographs (Figure 8c,d). Adequate adhesion throughout the porous construction indicates a good interaction between cells and scaffolds. Meanwhile, the higher cell density and greater amount of spread cells observed in alkalinity-modified P_80-20_, rather than P_80-20_, suggest the virtue of modified scaffolds for cell adhesion. Besides, quantitative results of cell adhesion further confirm that more living cells spread on alkalinity-modified P_80-20_ than the unmodified one (Figure 8b). These results reveal that alkaline modification heightens the applied value of PLA scaffolds in bone tissue engineering.

## 4. Conclusions

Interconnected porous PLA scaffolds were successfully developed via a SSE/water-soluble porogen leaching approach. The presence of PEG not only generated desirable processability of PLA/NaCl, but also facilitated the occurrence of an interconnected pore. In this scenario, removal of porogen consisted of NaCl (>75.00 wt %) and PEG (>1.25 wt %) permitted the construction of a three-dimensional porous architecture with high connectivity, covering a value of more than 97% and enhanced porosity outperforming 60%, assuring enough space to promote cell proliferation and growth. A significant merit in innumerable micropores and coarse surface topography throughout pore walls was favorable to the flow transport of nutrients and metabolic waste, and cell adhesion. Positively, compressive modulus varying from 85.7 to 207.4 MPa fell in the range of normal modulus of human trabecular bone (50–250 MPa). *In vitro* cell experiments showed that biocompatible PLA scaffolds promoted cell attachment after alkaline modification. As a whole, porous PLA scaffolds prepared by the SSE/porogen leaching approach integrated desired pore parameters with interconnected structures, good physical performances, as well as biological properties. Thus, our findings display that the SSE/porogen leaching could be fruitfully applied to high-performance porous scaffolds for the use of bone repair.

## Figures and Tables

**Table 1:** Composition formulation and theoretical porosity of ternary blends of poly(lactic acid) (PLA)/poly(ethylene glycol) (PEG)/NaCl.

	P_75-5_	P_75-10_	P_75-20_	P_80-20_	P_85-20_
**NaCl (wt %)**	75.00	75.00	75.00	80.00	85.00
**PEG (wt %)**	1.25	2.50	5.00	4.00	3.00
**PLA (wt %)**	23.75	22.50	20.00	16.00	12.00
**MR (wt %) ^1^**	5	10	20	20	20
**Theoretical porosity (%)**	65.66	67.55	71.29	76.28	81.62

^1^ MR wt % = *m*_PEG_/(*m*_PEG_ + *m*_PLA_).

## Supplementary Materials

Supplementary Materials can be found at www.mdpi.com/2073-4360/8/6/213/s1.
