# Supplementary material for: Engineering Porous Poly(lactic acid) Scaffolds with High Mechanical Performance via a Solid State Extrusion/Porogen Leaching Approach
Source: Polymers (Basel). 2016 May 31;8(6):213. doi: 10.3390/polym8060213 (PMC6432203; doi:10.3390/polym8060213)
Supplement: Supplementary file 1 [file polymers-08-00213-s001.pdf]

Supplementary Materials: Engineering Porous Poly(lactic acid) Scaffolds with High Mechanical Performance *via* a Solid State Extrusion/Porogen Leaching Approach

Hua-Mo Yin, Jing Qian, Jin Zhang, Zai-Fu Lin, Jian-Shu Li, Jia-Zhuang Xu and Zhong-Ming Li

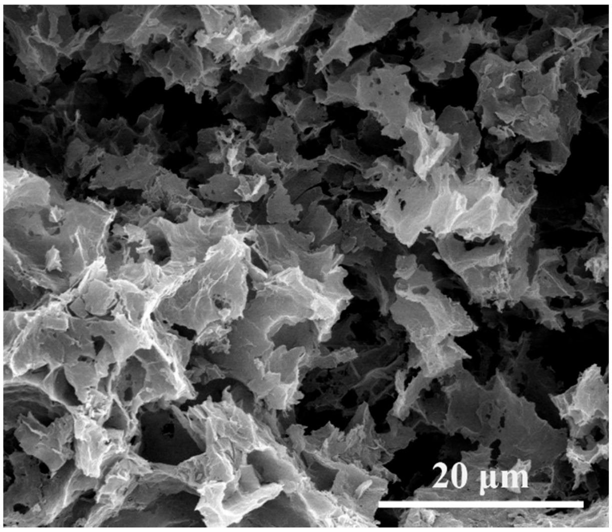

Figure S1. SEM image of fracture surface of scaffolds formed by $P_{85\text{-}20}$.

© 2016 by the authors; licensee MDPI, Basel, Switzerland. This article is an open access article distributed under the terms and conditions of the Creative Commons Attribution (CC-BY) license (http://creativecommons.org/licenses/by/4.0/).
